# Prevalence of Healthcare-Associated Infections in a Tertiary Hospital in Casablanca, Morocco, 2021

**DOI:** 10.7759/cureus.67171

**Published:** 2024-08-19

**Authors:** Soukaina Lyazidi, Mohamed Ouhadous, Mounir Arai, Khalid Zerouali, Houcine Barrou, Samira Hassoune

**Affiliations:** 1 Laboratory of Epidemiology, Faculty of Medicine and Pharmacy, University Hassan II, Casablanca, MAR; 2 Hospital Hygiene Department, Ibn Rochd University Hospital, Casablanca, MAR; 3 Nosocomial Infections Control Committee, Ibn Rochd University Hospital, Casablanca, MAR; 4 Microbiology Laboratory, Ibn Rochd University Hospital, Casablanca, MAR; 5 Surgical Intensive Care Unit, Ibn Rochd University Hospital, Casablanca, MAR; 6 Laboratory of Cellular and Molecular Pathology/Epidemiology and Histology of Cancerous Diseases Research, Faculty of Medicine and Pharmacy, University Hassan II, Casablanca, MAR

**Keywords:** acinetobacter baumanii, antimicrobial resistance, prevalence study, cross-infection, healthcare associated infections (hai)

## Abstract

Background: During the COVID-19 pandemic, healthcare professionals experienced an increased workload, which may have affected infection prevention and control (IPC) programs and consequently healthcare-associated infection (HAI) rates. The objective of this study was to estimate the prevalence of HAI in Ibn Rochd University Hospital Center (IRUHC) and identify associated factors.

Methods: A survey was conducted on November 30, 2021 at IRUHC, including all patients hospitalized for at least 48 hours. Data was collected using a questionnaire, and analyzed using SPSS IBM software version 16. The significance level was set at 0.05.

Results: Among 887 patients, the prevalence of HAI was 9.7% (7.7%; 11.6%). The highest prevalence was observed in intensive care units (ICUs) (44.2%). Nosocomial pneumonia was the most common site (26.8%). The main isolated microorganisms were *Acinetobacter baumannii* (18.0%) and *Escherichia coli* (16.0%). All *Acinetobacter baumannii* isolated strains were imipenem-resistant. The presence of HAI was significantly associated with the presence of an invasive medical device (p<0.001), a higher physical status score of American Society of Anesthesiologists (ASA) (p<0.001), and a longer hospital stay (p<0.001).

Conclusion : The emergence of imipenem-resistant *Acinetobacter baumannii* (IRAB) represents a serious therapeutic and epidemiological problem requiring the establishment of a system for monitoring the microbial environment and the application of strict hygiene measures.

## Introduction

Healthcare-associated infections (HAIs) continue to burden healthcare systems with excess morbidity, mortality, and additional financial cost [1]. According to World Health Organization (WHO), 7% of patients in acute-care hospitals in high-income countries and 15% in low- and middle-income countries will acquire at least one HAI during their hospital stay. On average,10% of affected patients will die from their HAI [2].

An HAI is an infection that is acquired by a patient during care delivery in a hospital or other healthcare facilities and was not present or incubating on admission [3]. These infections are mostly caused by microorganisms resistant to one or more commonly-used antibiotics [3]. They can be associated with devices used in medical procedures, such as catheters or ventilators. Common HAIs include central line-associated bloodstream infections (BSIs), catheter-associated urinary tract infections, ventilator-associated pneumonia, and surgical site infections (SSIs) [4].

Before the pandemic, HAI prevention and control interventions showed progress in reducing HAI rates and other adverse outcomes [5,6].

The COVID-19 pandemic has significantly affected healthcare systems globaly. Healthcare professionals experienced high levels of burnout during the pandemic due to increased workload [7,8]. This may have affected infection prevention and control (IPC) programs and consequently HAI rates. Data from the USA National Healthcare Safety Network (NHSN) showed significantly higher incidence of different types of HAI in 2021 compared to 2019 [9].

In the pandemic, from 2020 to 2021, Ibn Rochd University Hospital Center (IRUHC), Casablanca, was dedicated to providing care to critical cases of COVID-19 requiring the resuscitation or intensive care. Alongside their usual activities, health professionals working in the IRUHC participated in the care of COVID-19 patients.

The nosocomial infection control committee of IRUHC and the hospital hygiene service organized the fifth HAI point prevalence study (PPS) in the post-COVID-19 pandemic period. The objective of this study was to estimate the prevalence and identify associated factors of HAIs in IRUHC of Casablanca during the pandemic.

## Materials and methods

Study design and setting

A cross-sectional point prevalence survey was conducted on November 30th,2021 in three institutions of IRUHC, Casablanca, Morocco.

Study population

The study included all patients who were hospitalized for at least 48 hours in one of the IRUHC departments and who were present on the day of the survey.

Newborns who were less than two days old, patients admitted for emergency observation, patients in day hospitals and patients in dental centers were excluded from the study.

Data collection procedure

Data was collected using a questionnaire by residents and nurses who had received a training before the survey day. Data was collected from medical records with the support of the attending physician and nurse.

Variables

The questionnaire presented two sections. The first section included patients general information (age, gender, length of stay, immunosuppression, American Society of Anesthesiologists (ASA) score, presence of invasive medical device, surgical procedure, etc.). The second section was intended only for patients who had an HAI (site of infection, isolated germs, etc.).

The criteria for defining a healthcare-associated infection were based on definitions proposed by the WHO [10]. An HAI is defined as an infection acquired during a hospital stay and occurring more than 48 hours after admission. The diagnosis of respiratory infection was suggested in the presence of respiratory symptoms with at least two of the following signs appearing during hospitalization: cough, purulent sputum, new infiltration visible on chest X-ray and compatible with the diagnosis of infection. The diagnosis of SSI was suggested in the presence of any purulent discharge, surgical abscess or extensive cellulitis on the surgical site in the month following surgery. A positive urine culture (one or two species) with at least 105 bacteria/ml, with or without clinical symptoms led to the diagnosis of urinary tract infection. A vascular catheter infection has been suggested in the presence of inflammation, lymphangitis or purulent discharge at the catheter insertion site. The diagnosis of sepsis was evoked in the presence of fever or chills and at least one positive blood culture.

Statistical analysis

The SPSS IBM Version 16 software was used for statistical data analysis. Categorically measured variables were described using frequencies and percentages, and quantitative variables using means and standard deviation. The chi-squared test of independence was used to assess associations between categorically measured variables. The level of two-tailed significance was set at p ≤ 0.05.

Ethical consideration

The anonymity and confidentiality were respected. The identification of patients was done using a hospitalization number in order to collect the results of the bacteriological examinations.

## Results

Patients' characteristics

A total of 887 patients participated in this survey. 463 patients (54.2%) were male. The 0-14 age group was the most represented (22.4%). Among our patients, 68.8% were admitted for a medical diagnosis. 531 patients (60.2%) had a planned admission, and 22.6% first went to emergency department. 40% patients stayed more than seven days in the hospital. 288 patients (32.8%) were immunodepressed and 173 patients (19.5%) underwent surgery during their hospital stay. The main class of contamination in operated patients was class 1 corresponding to clean surgery (75.6%). More results are shown in Table 1.

**Table 1:** Characteristics of patients (n=887) ASA: American Society of Anesthesiologists; HAI: Healthcare-associated infection

Variable	n (%)
Sex	
Male	463 (54.2)
Female	391 (45.8)
Age groups (years)	
0-14	189 (22.4)
15-29	149 (17.7)
30-44	182 (21.6)
45-59	155 (18.4)
60-74	132 (15.6)
≥75	37 (4.4)
Provenance	
Planned admission	531 (60.2)
Emergency	199 (22.6)
Transferred	152 (17.2)
Immunodepressed	288 (32.8)
ASA score	
1	383 (47.9)
2	246 (30.8)
3	138 (17.3)
4	32 (4.0)
Length of stay (days)	
0-3	306 (35.0)
4-7	214 (24.5)
8-11	86 (9.9)
≥12	267 (30.6)
Admission diagnosis	
Medical	605 (68.8)
Urgent surgery	84 (9.6)
Elective surgery	165 (18.8)
Others	25 (2.8)
Surgical intervention	173 (19.5)
Classification of surgical wounds (n= 173)	
Clean	130 (75.6)
Clean-contaminated	20 (11.6)
Contaminated	13 (7.6)
Dirty or infected	9 (5.2)
Invasive medical device	
None	270 (30.4)
1	465 (52.4)
≥2	152 (17.2)
Antimicrobial treatment	446 (50.3)
Indication of antimicrobial	
Community-acquired infections	200 (22.5)
Prophylactic indication	187 (21.1)
HAIs	76 (8.6)

On the survey day, 69.6% of patients had at least one invasive medical device. A peripheral venous catheter (PVC), urinary catheter, central venous catheter (CVC), endotracheal tube, nasogastric tube, tracheostomy tube, and arterial catheter were found respectively in 64.9%, 14.0%, 8.0%, 3.5%, 2.6%, 1.1% and 0.6% of patients.

Antimicrobial prescription

Among our patients, 446 (50.3%) were receiving at least one antimicrobial drug. These latter were prescribed for a community-acquired infection in 22.5% of patients, for a prophylactic indication in 21.1% and for a HAI in 8.6% (Table 1).

The main antibiotics prescribed were penicillin A-beta-lactamase inhibitors association, third-generation cephalosporins, aminoglycosides and fluoroquinolones (33.0%, 26.5%, 15.2% and 13.7% of total patients receiving antimicrobial, respectively).

Prevalence of HAIs

86 patients had at least one HAI, corresponding to a prevalence of 9.7% with a 95% confidence interval of (7.7%; 11.6%).

The highest prevalence of HAI was observed in intensive care units (ICUs) (44.2%), followed by hemato-oncology (18.3 %) and pediatric departments (13.7%).

HAI sites

Nosocomial pneumonia was the most common site representing 26.8% of HAIs followed by SSI (15.5%) and BSI (14.4%) (Table 2).

**Table 2:** Characteristics of HAIs HAI: Healthcare-associated infection; SSI: Surgical site infection; BSI: Bloodstream infection; MDR: Multidrug resistant; IRAB: Imipenem-resistant *Acinetobacter baumannii*; ESBL: Extended spectrum beta lactamase; IRPA: Imipenem-resistant *Pseudomonas aeruginosa*; CRE: Carbapenem-resistant Enterobacteriaceae; CRPA: Ceftazidime-resistant *Pseudomonas aeruginosa*

Variable	n (%)
Sites of infection (n=97)	
Pneumonia	26 (26.8)
SSI	15 (15.5)
BSI	14 (14.4)
Urinary tract infection	11 (11.3)
Skin and soft tissue infection	10 (10.3)
Nosocomial meningitis	9 (9.3)
Catheter-associated infection	4 (4.1)
Ascitic fluid infection	3 (3.1)
Other	5 (5.2)
Isolated microorganisms (n=50)	
Acinetobacter baumannii	9 (18.0)
Escherichia coli	8 (16.0)
Coagulase negative Staphylococcus	7 (14.0)
Klebsiella pneumoniae	6 (12.0)
Pseudomonas aeroginosa	6 (12.0)
Enterobacter cloacae	5 (10.0)
Staphylococcus aureus	2 (4.0)
Serratia sp	1 (2.0)
Acinobacter sp	1 (2.0)
Pseudomonas sp	1 (2.0)
Other	4 (8.0)
MDR bactreria (n=21)	
IRAB	9 (42.9)
ESBL producers	8 (38.1)
IRPA	4 (19.0)
CRE	1 (4.8)
CRPA	1 (4.8)

Isolated germs and multidrug resistant bacteria

Germs were isolated in 50 patients (58.1%) with HAI. The main microorganisms were *Acinetobacter baumannii* (18.0%) and *Escherichia coli* (16.0%). Multidrug resistant (MDR) bacteria represented 42.0% of the isolated germs (21/50). All *Acinetobacter baumannii* isolated strains were imipenem-resistant and represented 42.9% of MDR bacteria. Extended-spectrum beta-lactamase (ESBL)-producing Enterobacteriaceae represented 38.1% of MDR bacteria (Table 2).

HAIs' associated factors

The presence of HAI was significantly associated with the presence of invasive medical device (p<0.001), the presence of two medical devices or more (p<0.001), a higher ASA score (p<0.001) and a longer hospital stay (p<0.001) (Table 3).

**Table 3:** HAIs' associated factors (n=887) HAI: Healthcare-associated infection; ASA: American Society of Anesthesiologists p-value threshold for significance =0.05 ***p<0.001

Variables	HAI n (%)	p value
Age groups		0.538
0-14	23 (12.2)	
15-29	13 (8.8)	
30-44	14 (7.7)	
45-59	10 (6.5)	
60-74	13 (9.8)	
≥75	3 (8.1)	
ASA score		<0.001***
1	16 (4.2)	
2	36 (14.6)	
3	16 (11.6)	
4	14 (43.8)	
Length of stay (days)		<0.001***
0-3	7 (2.3)	
4-7	15 (7.0)	
8-11	11 (12.9)	
≥ 12	52 (19.5)	
Invasive medical device		<0.001***
Yes	80 (13.1)	
No	6 (2.2)	
Number of Invasive medical devices		<0.001***
None	3 (1.1)	
1	43 (9.3)	
≥2	40 (26.3)	

SSI-associated factors

The presence of SSI was significantly associated with the class of contamination (p = 0.006) and not taking antibiotic prophylaxis (p = 0.014) (Table 4).

**Table 4:** SSI-associated factors in operated patients (n=173) ASA: American Society of Anesthesiologists; SSI: Surgical site infection p-value threshold for significance=0.05 *p<0.05; **p<0.01

Variables	SSI n (%)	p-value
Gender		0.411
Male	4 (5.3)	
Female	2 (2.2)	
Score ASA		0.332
1-2	4 (3.1)	
3-4	2 (6.5)	
Classification of surgical wounds		0.006**
Clean/Clean-contaminated	3 (2.0)	
Contaminated/Dirty	4 (18.2)	
Urgent surgery		0.104
Yes	5 (7.8)	
No	2 (1.9)	
Antibiotic prophylaxis		0.014*
Yes	1 (0.9)	
No	6 (9.0)	

## Discussion

The current study is the fifth HAI PPS carried out in the IRUHC, Casablanca, Morocco. The prevalence of HAI was 10.3% in the PPS of 2014, 5.4% in the PPS of 2017 and 9.7% in the present study conducted in post-COVID-19 pandemic period [11,12].

The COVID-19 pandemic has placed substantial burdens on healthcare systems and hospitals worldwide. Healthcare workers’ burnout, higher than usual hospitalizations and shortages in equipment might have impacted infection prevention efforts and common HAI rates. A previous study of US hospitals has shown that certain HAIs increased during the pandemic [13]. Similar results were reported by a study conducted in seven resource-limited countries and a Dutch study [14,15]. Conversely, other studies has shown that the pandemic has led to enhanced infection prevention efforts and a reduction in HAI rates during the ongoing pandemic [16-18].

The highest prevalence of HAIs in our study was observed in ICUs, which is consistent with the local PPS of 2017 and other studies [12,19-21]. Hemato-oncology departments came in second position in our study with a HAI prevalence of 18.3%. This may be due to the high use of invasive devices and the high frequency of immunocompromised patients in these departments. In fact, neutropenia, chemotherapy and central venous catheterization have been identified as risk factors implicated in the occurrence of HAI [22,23].

Similarly to results reported in the local PPS of 2017, nosocomial pneumonia, SSI and BSI were the most common sites of HAI in this study [12]. However, in the local PPS of 2014 urinary tract infection was the main HAI [11]. SSI and BSI were the most common HAIs reported in African studies according to a systematic review [24].

High rates of SSI were reported by different studies [20,25-27]. Appropriate perioperative antibiotic prophylaxis is an important component of SSI prevention. Centers for Disease Control and Prevention Guideline for the Prevention of Surgical Site Infection (2017) included three measures related to perioperative antibiotic prophylaxis: timing, drug appropriateness and drug discontinuation after surgery [28].

The main microorganism isolated in our patients with HAI was *Acinetobacter baumannii*, and all isolated strains were imipenem-resistant. *Acinetobacter baumannii* is an opportunistic human pathogen that predominantly infects critically ill patients. It is considered a global threat for the healthcare system, mainly due to its tendency to acquire multidrug resistance phenotypes at high rates [29,30]. Infections caused by *Acinetobacter baumannii* account for nearly 2% of all HAIs in the United States and Europe [31,32]. However, this proportion is twice as high in Asia and the Middle East [32]. Although infection rates are lower than those caused by other Gram-negative pathogens, globally, nearly 45% of all isolates are considered to be MDR, with a proportion as high as 70% in Latin America and the Middle East [29]. These MDR proportions are nearly four times higher than those observed for other Gram-negative pathogens, such as *Pseudomonas aeruginosa* and *Klebsiella pneumoniae* [29]. Recently, carbapenem-resistant *Acinetobacter baumannii* has been added to the WHO published list of bacteria for which new antibiotics are urgently needed to address growing global resistance [33].

The prevalence of antimicrobial use slightly increased from 2017 to 2021, from 46.5% to 50.3%, respectively. A multicenter Ethiopian study conducted in 2021 reported a higher prevalence of Antibiotic use of 63.8% [34]. These results has practical and policy implications for strengthening antimicrobial stewardship programs and promoting rational antibiotics use.

In our study, the main antibiotics prescribed were penicillin A-beta-lactamase inhibitors association, third-generation cephalosporins, aminoglycosides and fluoroquinolones. Similar results were reported by a global PPS conducted in 2015, including 53 countries from low, high and middle-income countries, with the highest prevalence of antibiotic prescription being in Africa [35].

In our study, Identified risk factors for HAIs were longer hospital stay, the presence of an invasive medical device, and high ASA score. Similar results were reported in the two previous local PPS [11,12]. Regarding factors significantly associated with SSI, we have identified the class of contamination (p=0.006) and not taking antibiotic prophylaxis (p=0.014). Similar results were reported by other studies [36-38]. Other risk factors have been reported in the literature such as the duration of surgery, length of preoperative hospital stay and ASA score [36-38].

Robust surveillance system is essential to the implementation of effective interventions and to assess the effectiveness of IPC programs. As described by the WHO, the core components of an effective IPC program include: establishing guidelines; supporting education and training; establishing HAI surveillance; using multimodal strategies; monitoring and evaluation of IPC practices; adequate staffing according to workload; adequate availability of materials and equipment for IPC [39].

This study has some limitations. It is a cross-sectional study, which is likely to underestimate the burden of HAIs. However, point prevalence surveys are cost-effective and allow the collection of a greater amount of information in a shorter period of time than incidence studies. Underestimation of HAI prevalence could also be a result of information bias, which may be due to interobserver variation in case definition or failure to identify HAIs due to limited or absent microbiology results for some patients. Another limitation is that the sample is not statistically representative of the general population, as our sample was drawn from a specific population hospitalized in a tertiary hospital, which may be vulnerable. However, the sample size was quite large, with 887 patients included in the analysis. 

## Conclusions

The emergence of imipenem-resistant *Acinetobacter baumannii* (IRAB) represents a serious therapeutic and epidemiological problem requiring the establishment of a system for monitoring the microbial environment and the application of strict hygiene measures.

Following the results of this study, the hospital hygiene department of the IRUHC set up an improvement program including a set of operational objectives. The first objective is to provide training and awareness in hospital hygiene for medical and paramedical staff and students. Continuous monitoring of the incidence of HAIs and cases of MDR bacteria infections must be ensured in all hospital departments. In addition, daily monitoring of waste management, bio-cleaning, and laundry management must be ensured to control the risk related to the environment.

## References

[1] Liu JY, Dickter JK (2020). Nosocomial infections: a history of hospital-acquired infections. Gastrointest Endosc Clin N Am.

[2] (2024). Global report on infection prevention and control: executive summary. https://www.who.int/publications/i/item/global-report-on-infection-prevention-and-control--executive-summary.

[3] (2024). Health care without avoidable infections: the critical role of infection prevention and control. https://www.who.int/publications/i/item/WHO-HIS-SDS-2016.10.

[4] (2024). About HAIs. https://www.cdc.gov/healthcare-associated-infections/about/index.html.

[5] DiDiodato G, McArthur L (2016). Evaluating the effectiveness of an antimicrobial stewardship program on reducing the incidence rate of healthcare-associated clostridium difficile infection: a non-randomized, stepped wedge, single-site, observational study. PLoS One.

[6] Schreiber PW, Sax H, Wolfensberger A, Clack L, Kuster SP (2018). The preventable proportion of healthcare-associated infections 2005-2016: systematic review and meta-analysis. Infect Control Hosp Epidemiol.

[7] Galanis P, Vraka I, Fragkou D, Bilali A, Kaitelidou D (2021). Nurses' burnout and associated risk factors during the COVID-19 pandemic: a systematic review and meta-analysis. J Adv Nurs.

[8] Raudenská J, Steinerová V, Javůrková A, Urits I, Kaye AD, Viswanath O, Varrassi G (2020). Occupational burnout syndrome and post-traumatic stress among healthcare professionals during the novel coronavirus disease 2019 (COVID-19) pandemic. Best Pract Res Clin Anaesthesiol.

[9] (2024). COVID-19 impact on healthcare-associated infections. https://www.cdc.gov/healthcare-associated-infections/php/data/covid-impact.html.

[10] World Health Organization (2024). Prévention des infections nosocomiales : guide pratique, 2nd. ed. https://iris.who.int/bitstream/handle/10665/69751/WHO_CDS_CSR_EPH_2002.12_fre.pdf?sequence=1&isAllowed=y.

[11] Hassoune S, Ouhadous M, El Bouri H, Nani S, Barrou H (2016). Prevalence des infections associees aux soins au centre hospitalier universitaire de Casablanca, Maroc, 2014. Rev Epidemiol Sante Publique.

[12] Lyazidi S, Arai M, Ouhadous M, Zerouali K, Barrou L, Hassoune S (2022). Prevalence des infections nosocomiales au sein du centre hospitalier universitaire Ibn Rochd de Casablanca. Rev Maroc Sante Publique.

[13] Baker MA, Sands KE, Huang SS (2022). The impact of coronavirus disease 2019 (COVID-19) on healthcare-associated infections. Clin Infect Dis.

[14] Rosenthal VD, Myatra SN, Divatia JV (2022). The impact of COVID-19 on health care-associated infections in intensive care units in low- and middle-income countries: International Nosocomial Infection Control Consortium (INICC) findings. Int J Infect Dis.

[15] Verberk JD, van der Kooi TI, Kampstra NA (2023). Healthcare-associated infections in Dutch hospitals during the COVID-19 pandemic. Antimicrob Resist Infect Control.

[16] Wee LE, Conceicao EP, Tan JY (2021). Unintended consequences of infection prevention and control measures during COVID-19 pandemic. Am J Infect Control.

[17] Thaprawat P, Greene MT, Saint S, Kasatpibal N, Fowler KE, Apisarnthanarak A (2022). Status of hospital infection prevention practices in Thailand in the era of COVID-19: results from a national survey. Am J Infect Control.

[18] Rong R, Lin L, Yang Y (2023). Trending prevalence of healthcare-associated infections in a tertiary hospital in China during the COVID-19 pandemic. BMC Infect Dis.

[19] Mpinda-Joseph P, Anand Paramadhas BD, Reyes G (2019). Healthcare-associated infections including neonatal bloodstream infections in a leading tertiary hospital in Botswana. Hosp Pract (1995).

[20] Saleem Z, Hassali MA, Godman B, Hashmi FK, Saleem F (2019). A multicenter point prevalence survey of healthcare-associated infections in Pakistan: findings and implications. Am J Infect Control.

[21] Phu VD, Wertheim HF, Larsson M (2016). Burden of hospital acquired infections and antimicrobial use in Vietnamese adult intensive care units. PLoS One.

[22] Fares S, Lamchahab M, Cherkaoui S, Zerouali K, Srhier Z, Quessar A (2021). Surveillance des infections associees aux soins dans une unite d’hematologie adulte a Casablanca. Rev Maroc Sante Publique.

[23] Mellouli A, Chebbi Y, Belloumi D (2021). Prevalence of healthcare-associated infections at a Tunisian onco-hematology ward. Tunis Med.

[24] Abubakar U, Amir O, Rodríguez-Baño J (2022). Healthcare-associated infections in Africa: a systematic review and meta-analysis of point prevalence studies. J Pharm Policy Pract.

[25] Abubakar U (2020). Point-prevalence survey of hospital acquired infections in three acute care hospitals in Northern Nigeria. Antimicrob Resist Infect Control.

[26] Labi AK, Obeng-Nkrumah N, Owusu E (2019). Multi-centre point-prevalence survey of hospital-acquired infections in Ghana. J Hosp Infect.

[27] Raka L, Spahija G, Gashi-Gecaj A (2019). Point prevalence survey of healthcare-associated infections and antimicrobial use in Kosovo hospitals. Infect Dis Rep.

[28] Berríos-Torres SI, Umscheid CA, Bratzler DW (2017). Centers for Disease Control and Prevention guideline for the prevention of surgical site infection, 2017. JAMA Surg.

[29] Giammanco A, Calà C, Fasciana T, Dowzicky MJ (2017). Global assessment of the activity of tigecycline against multidrug-resistant Gram-negative pathogens between 2004 and 2014 as part of the tigecycline evaluation and surveillance trial. mSphere.

[30] Rolain JM, Diene SM, Kempf M, Gimenez G, Robert C, Raoult D (2013). Real-time sequencing to decipher the molecular mechanism of resistance of a clinical pan-drug-resistant Acinetobacter baumannii isolate from Marseille, France. Antimicrob Agents Chemother.

[31] Magill SS, Edwards JR, Bamberg W (2014). Multistate point-prevalence survey of health care-associated infections. N Engl J Med.

[32] Lob SH, Hoban DJ, Sahm DF, Badal RE (2016). Regional differences and trends in antimicrobial susceptibility of Acinetobacter baumannii. Int J Antimicrob Agents.

[33] (2024). WHO publishes list of bacteria for which new antibiotics are urgently needed. https://www.who.int/news/item/27-02-2017-who-publishes-list-of-bacteria-for-which-new-antibiotics-are-urgently-needed.

[34] Fentie AM, Degefaw Y, Asfaw G, Shewarega W, Woldearegay M, Abebe E, Gebretekle GB (2022). Multicentre point-prevalence survey of antibiotic use and healthcare-associated infections in Ethiopian hospitals. BMJ Open.

[35] Versporten A, Zarb P, Caniaux I (2018). Antimicrobial consumption and resistance in adult hospital inpatients in 53 countries: results of an internet-based global point prevalence survey. Lancet Glob Health.

[36] Zine K, Hassoune S, Fahmi Y (2014). Surveillance des infections du site operatoire en chirurgie viscerale au centre hospitalier universitaire Ibn Rochd de Casablanca. Rev Maroc Sante Publique.

[37] Carvalho RL, Campos CC, Franco LM, Rocha AM, Ercole FF (2017). Incidence and risk factors for surgical site infection in general surgeries. Rev Lat Am Enfermagem.

[38] Shrestha S, Wenju P, Shrestha R, Karmacharya RM (2016). Incidence and risk factors of surgical site infections in Kathmandu University Hospital, Kavre, Nepal. Kathmandu Univ Med J (KUMJ).

[39] World Health Organization (2024). Minimum requirements for infection prevention and control programmes. https://iris.who.int/bitstream/handle/10665/330080/9789241516945-eng.pdf?sequence=1.

